# Targeted Prostate Biopsy: How, When, and Why? A Systematic Review

**DOI:** 10.3390/diagnostics14171864

**Published:** 2024-08-26

**Authors:** Giacomo Rebez, Maria Barbiero, Franco Alchiede Simonato, Francesco Claps, Salvatore Siracusano, Rosa Giaimo, Gabriele Tulone, Fabio Vianello, Alchiede Simonato, Nicola Pavan

**Affiliations:** 1 Urology Unit, Dipartimento Chirurgico Area Isontina, Azienda Sanitaria Universitaria Giuliano Isontina, 34170 Gorizia, Italy; giacomorebez@gmail.com (G.R.); fabio.vianello@asugi.sanita.fvg.it (F.V.); 2 Department of Medical, Surgical and Health Science, Urology Clinic, University of Trieste, 34100 Trieste, Italy; maria.barbiero93@gmail.com (M.B.); claps.francesco@gmail.com (F.C.); 3 Medical School, University of Genoa, 16100 Genoa, Italy; francoalchiede@gmail.com; 4 Department of Urology, University of L’Aquila, 67100 L’Aquila, Italy; salvatore.siracusano@univaq.it; 5 Urology Clinic, Department of Precision Medicine in Medical, Surgical and Critical Care, University of Palermo, 90127 Palermo, Italy; rosa.giaimo@policlinico.pa.it (R.G.); gabriele.tulone@policlinico.pa.it (G.T.); alchiede.simonato@unipa.it (A.S.)

**Keywords:** targeted prostate biopsy, multiparametric MRI, transrectal ultrasound, diagnostic accuracy, prostate cancer detection

## Abstract

Objective: Prostate cancer, the second most diagnosed cancer among men, requires precise diagnostic techniques to ensure effective treatment. This review explores the technological advancements, optimal application conditions, and benefits of targeted prostate biopsies facilitated by multiparametric magnetic resonance imaging (mpMRI). Methods: A systematic literature review was conducted to compare traditional 12-core systematic biopsies guided by transrectal ultrasound with targeted biopsy techniques using mpMRI. We searched electronic databases including PubMed, Scopus, and Web of Science from January 2015 to December 2024 using keywords such as “targeted prostate biopsy”, “fusion prostate biopsy”, “cognitive prostate biopsy”, “MRI-guided biopsy”, and “transrectal ultrasound prostate biopsy”. Studies comparing various biopsy methods were included, and data extraction focused on study characteristics, patient demographics, biopsy techniques, diagnostic outcomes, and complications. Conclusion: mpMRI-guided targeted biopsies enhance the detection of clinically significant prostate cancer while reducing unnecessary biopsies and the detection of insignificant cancers. These targeted approaches preserve or improve diagnostic accuracy and patient outcomes, minimizing the risks associated with overdiagnosis and overtreatment. By utilizing mpMRI, targeted biopsies allow for precise targeting of suspicious regions within the prostate, providing a cost-effective method that reduces the number of biopsies performed. This review highlights the importance of integrating advanced imaging techniques into prostate cancer diagnosis to improve patient outcomes and quality of life.

## 1. Introduction

Prostate cancer, ranked as the second most frequently diagnosed cancer among men, poses a significant public health concern due to its substantial mortality rate [1]. The early and precise diagnosis of low and intermediate-risk prostate cancer is crucial for the success of therapeutic interventions, underpinning the necessity for advancements in diagnostic techniques. Traditionally, the transrectal, ultrasonographically guided, 12-core systematic biopsy has been the go-to technique for diagnosing prostate cancer. However, this conventional approach is not without its limitations, as it is prone to both overdiagnosis and underdiagnosis of the disease [2]. The inherent shortcomings of systematic biopsies have necessitated a paradigm shift in the diagnostic landscape.

Multiparametric magnetic resonance imaging (mpMRI) plays a growing and pivotal role in prostate cancer diagnosis [3]. Utilizing MRI data, prostate biopsy cores can be precisely directed to suspicious regions within the prostate. Evidence from systematic reviews and randomized controlled trials (RCTs) challenges the conventional diagnostic approach of a 10–12-core transrectal ultrasound (TRUS)-guided prostate biopsy in men with elevated prostate-specific antigen (PSA) [4].

The advent of targeted biopsies, facilitated by mpMRI, offers several potential benefits. Firstly, it preserves or enhances the detection rates of clinically significant diseases while simultaneously reducing the number of biopsies performed. This reduction in biopsies, particularly on a smaller cohort of individuals, holds promise for cost-effectiveness in diverse healthcare settings. Furthermore, targeted biopsies contribute to a decrease in the detection of clinically insignificant diseases, mitigating the risk of overtreatment and its associated consequences.

This review seeks to delve deeper into the intricacies of targeted prostate biopsy, exploring the ‘how’, ‘when’, and ‘why’ as illuminated by current literature. It investigates the technological advancements that make targeted biopsies possible, the circumstances under which this approach should be considered, and the compelling rationale behind this shift in prostate cancer diagnosis.

This exploration will illuminate the nuances of the procedure, provide insights into its optimal timing, and underscore its pivotal role in mitigating the issues of overdiagnosis and underdiagnosis, thereby ensuring improved patient outcomes and quality of life.

## 2. Materials and Methods

### 2.1. Data Collection and Study Selection

A systematic literature review was conducted to identify relevant studies addressing prostate biopsy techniques. Three electronic databases (PubMed, Scopus, and Web of Science) were searched from January 2015 to December 2024 using the following keywords: “targeted prostate biopsy”, “fusion prostate biopsy”, “cognitive prostate biopsy”, “MRI-guided biopsy”, and “transrectal ultrasound prostate biopsy”. Inclusion criteria encompassed studies comparing different biopsy methods, including in-bore MRI target biopsy, MRI-TRUS fusion biopsy, cognitive registration TRUS biopsy, and standard transrectal ultrasound-guided biopsy.

### 2.2. Screening and Eligibility Criteria

Studies were included if they provided data on diagnostic outcomes, complications, and comparisons between different prostate biopsy techniques. The exclusion criteria were as follows: reviews or editor letters and single case reports; non-English language publications; studies without consistent information on biopsy protocol and studies with insufficient or unconfirmed information. The eligibility process was performed independently by two reviewers, and any discrepancies were resolved through discussion.

### 2.3. Data Extraction and Synthesis

Data extraction included study characteristics, patient demographics, biopsy techniques, diagnostic outcomes, and complications. A standardized Excel (Version 16) form was used for data extraction, and the results were synthesized to address specific research questions regarding the diagnostic precision and safety of each biopsy method.

### 2.4. Quality Assessment

This review adheres to the Preferred Reporting Items for Systematic Reviews and Meta-Analyses (PRISMA) guidelines. The selection process, data extraction, and quality assessment with the Newcastle–Ottawa Scale (NOS) were documented to ensure transparency and reproducibility.

### 2.5. Potential Bias and Limitations

Possible sources of bias were identified; publication bias and selective reporting were acknowledged. To minimize these risks, we implemented a dual-review process wherein two independent authors (GR and NP) reviewed the papers. This approach helps to reduce subjective bias and ensure a more balanced and accurate assessment of the included studies.

## 3. Results

**Figure 1:**
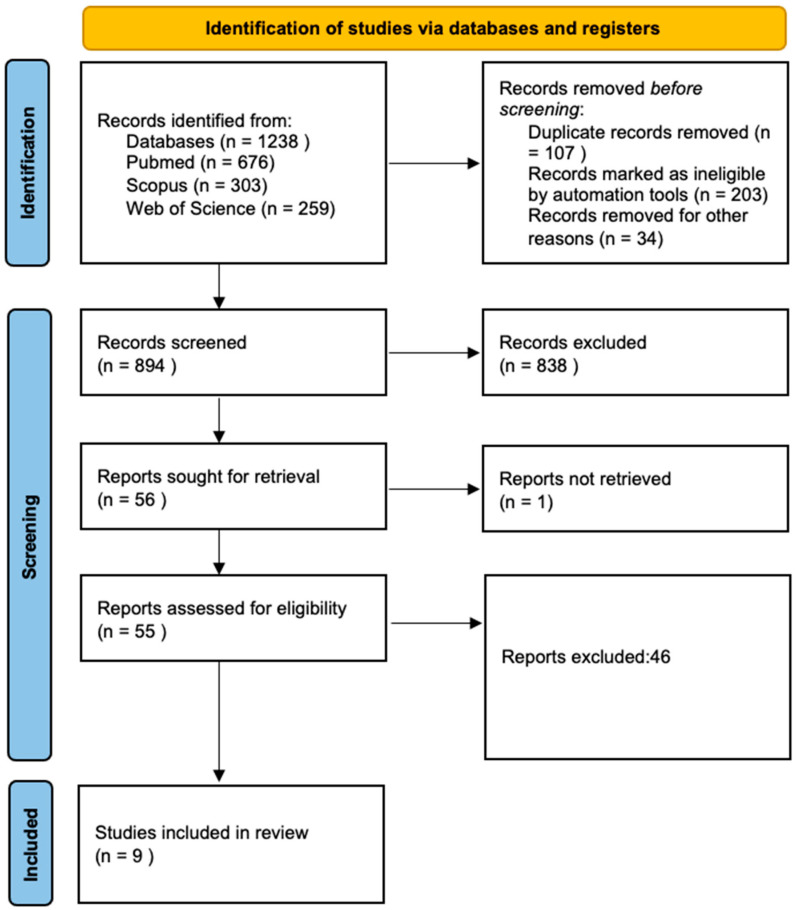
PRISMA flowchart.

Using these search criteria, an initial selection of 1238 articles was considered. After the exclusion of case series, review articles, and articles without biopsy protocol, we narrowed down to 894 studies that were selected for abstract screening. Finally, after the removal of duplicates, conference proceedings, abstracts, and non-English texts, 56 abstracts were reviewed. After a comprehensive review process, 9 full-text articles were included in this review, in accordance with the aforementioned inclusion criteria. A PRISMA flowchart is represented in Figure 1.

Comparative analysis of MRI-targeted biopsy techniques revealed distinct approaches, including in-bore mpMRI, mpMRI-TRUS fusion, and cognitive registration TRUS-targeted biopsies. Each method offers unique advantages in enhancing diagnostic precision, detailed in Table 1, summarizing their methodologies, benefits, and limitations. In 2016, Baco et al. conducted a study with 175 biopsy-naive patients, utilizing a targeted biopsy approach for palpable or TRUS-suspicious lesions, coupled with a 12-core biopsy if needed, defining clinically significant prostate cancer based on maximum cancer core length (MCCL) criteria [5]. Pepe et al. (2016) engaged 200 patients in saturation transperineal prostate biopsy, employing targeted magnetic resonance imaging (mpMRI) and transrectal ultrasound (TRUS) fusion-guided biopsies along with cognitive transperineal biopsies, defining significance by Gleason score and positive cores [6]. Porpiglia et al. (2017) investigated 107 biopsy-naive individuals, incorporating a biopsy definition based on a Gleason score > 7 or maximum core cancer length > 5 mm [7]. Lastly, Kasivisvanathan et al. (2018) explored 252 biopsy-naive patients, focusing on the detection of Gleason 7 (3 + 4) and higher prostate cancers. Overall targeted biopsies, guided by mpMRI, demonstrated a higher detection rate of clinically significant prostate cancer (CSPC) compared to systematic biopsies [8].

In-bore MRI biopsy demonstrates a significantly higher target-specific cancer detection rate compared to fusion biopsy, especially for PI-RADS category 4 or 5 lesions [9].

Both transrectal and transperineal biopsy approaches show similar diagnostic accuracy for prostate cancer. Transperineal biopsies demonstrate better capabilities for detecting apical and anterior prostate cancers, coupled with lower complication rates.

### Clinical Trial

The PREVENT trial is a multicenter randomized trial that analyzed the infectious complications of transperineal prostate biopsy without antibiotic prophylaxis compared with transrectal biopsy with targeted prophylaxis. The study involved 658 biopsy-naive participants. The primary outcome, post-biopsy infection, occurred in zero participants with transperineal biopsy versus four (1.4%) with transrectal biopsy (*p* = 0.059). Cancer detection rates were similar (53% transperineal vs. 50% transrectal), and other complications were low and comparable. Although transperineal biopsy caused slightly more periprocedural pain, it was tolerable, did not compromise cancer detection, and did not result in infectious complications. Transrectal biopsy with targeted prophylaxis achieved similar infection rates but requires rectal cultures and careful antibiotic management [10].

PROMIS trial, a multi-center, paired-cohort study, aimed to evaluate the diagnostic accuracy of mpMRI and transrectal ultrasound-guided biopsy compared to a gold-standard reference template mapping biopsy. Inclusion criteria involved men (*n* = 576) with a PSA < 15 ng/mL and no previous biopsy history. Mapping biopsy results revealed that 71% of men had cancer, with 40% exhibiting clinically significant prostate cancer (Gleason score ≥ 4 + 3 or maximum cancer length ≥ 6 mm). In the context of clinically significant disease, mpMRI demonstrated higher sensitivity (93%) compared to transrectal ultrasound-guided biopsy (48%), though with lower specificity (41% for mpMRI; 96% for transrectal ultrasound-guided biopsy). These findings shed light on the comparative strengths and weaknesses of mpMRI and transrectal ultrasound-guided biopsy in diagnosing clinically significant prostate cancer [11].

The PRECISION trial enrolled 500 men with a clinical suspicion of prostate cancer who had not previously undergone a prostate biopsy. The trial compared MRI with or without a targeted biopsy against the standard transrectal ultrasound-guided biopsy. In the MRI group, men underwent a targeted biopsy if there was suspicion of prostate cancer on imaging and refrained from biopsy if the MRI yielded negative results. The primary outcome of this randomized clinical trial was the diagnosis of clinically significant prostate cancer. In the MRI-targeted biopsy group, 28% had a negative MRI and thus avoided biopsy. Among those undergoing targeted biopsy, 38% were diagnosed with clinically significant cancer, compared to 26% in the transrectal ultrasound-guided biopsy group (*p* = 0.005). Additionally, the MRI-targeted biopsy group exhibited fewer cases of clinically insignificant prostate cancer compared to the transrectal ultrasound-guided biopsy group [8].

2022 NEJM GÖTEBORG-2 spanning 37,887 men aged 50–60, participants were invited for a screening involving PSA and MRI, followed by targeted biopsy only. Of the 17,980 participants, 66 individuals (0.6%) in the experimental group were diagnosed with clinically insignificant prostate cancer, in contrast to 72 participants (1.2%) in the reference group. This marked a notable difference of −0.7 percentage points (95% CI −1.0 to −0.4), resulting in a relative risk of 0.46 (95% CI, 0.33 to 0.64). Additionally, the relative risk of clinically significant prostate cancer in the experimental group, when compared with the reference group, stood at 0.81 (95% CI, 0.60 to 1.1) [12].

## 4. Discussion

The MRI-TRUS fusion targeted biopsy method leverages cutting-edge software that combines MRI and TRUS (transrectal ultrasound) images, allowing for direct biopsies of MRI-identified lesions. Cognitive registration TRUS-targeted biopsies, on the other hand, entail a distinct approach. Before the biopsy, the MRI images are meticulously reviewed and assessed to cognitively guide the biopsy procedure using TRUS guidance. This method capitalizes on the expertise of the clinician to pinpoint MRI-identified lesions during the biopsy, ensuring precision [13].

In comparing MRI–US Fusion Biopsy (MRI-US FB) and Cognitive Biopsy (CB), a systematic review and meta-analysis involving 1714 men with MRI-identifiable lesions showed a trend toward improved prostate cancer detection rates with MRI-US FB, but the difference was not statistically significant. The odds ratios for overall and clinically significant cancer detection were 1.11 and 1.13, respectively. Moderate heterogeneity was observed but did not reach significance [14]. Moreover, in a separate analysis comparing Transperineal Software-Assisted Fusion Biopsy (saFB) and Cognitive Fusion Biopsies (cFB), the meta-analysis included 2112 cases. The findings indicated no substantial difference in the detection rates of clinically significant prostate cancer between saFB and cFB (OR 1.01). The study emphasized the absence of conclusive evidence supporting the superiority of saFB over cFB. Operator experience and software availability were identified as key factors influencing the choice between the two fusion techniques [15].

In a systematic review, both transperineal biopsy and transrectal prostate biopsy methods demonstrated comparable diagnostic accuracy for prostate cancer. However, the transperineal approach exhibited a significantly lower risk of fever and rectal bleeding [16]. Conversely, a meta-analysis incorporating data from various studies between April 2000 and August 2014 found no significant differences in prostate cancer detection rates between transperineal and transrectal approaches. Notably, there were no significant variations in abnormal digital rectal examination findings, serum PSA levels, Gleason scores, prostate volume, or relevant complications. However, the meta-analysis suggested that, in terms of pain relief and additional anesthesia, the transrectal prostate needle biopsy may be relatively preferable compared to the transperineal approach [17]. Similar results are provided by the PREVENT trial, a multicenter randomized trial that analyzed the infectious complications of transperineal prostate biopsy without antibiotic prophylaxis, which were compared with transrectal biopsy with targeted prophylaxis. The study involved 658 biopsy-naive participants. The primary outcome, post-biopsy infection, occurred in zero participants with transperineal biopsy versus four (1.4%) with transrectal biopsy (*p* = 0.059). Cancer detection rates were similar (53% transperineal vs. 50% transrectal), and other complications were low and comparable. Although transperineal biopsy caused slightly more periprocedural pain, it was tolerable, did not compromise cancer detection, and did not result in infectious complications. Transrectal biopsy with targeted prophylaxis achieved similar infection rates but requires rectal cultures and careful antibiotic management [10].

Collectively, these insights suggest that both transperineal and transrectal biopsy approaches for targeted biopsy offer similar diagnostic accuracy for prostate cancer. The choice between these methods may hinge on factors such as the specific regions of concern, patient tolerance for potential complications, and preferences regarding pain relief and anesthesia.

Notably, a staggering 95% of prostate biopsies presently utilize standard transrectal ultrasound guidance. However, inherent limitations in this method are underscored, emphasizing that while it provides a contour view of the prostate, it falls short in visualizing lesions effectively [18].

Moreover, the inadequacies of transrectal ultrasound guidance extend to the potential oversight of anterior and apical lesions, contributing to the risk of missing tumors in up to one-third of cases [19]. Data indicate that 2–7% of patients may experience urinary tract infections, epididymitis, orchitis, prostatitis, and, in severe cases, sepsis [20].

In this retrospective study comparing in-bore and fusion MRI-targeted prostate biopsies for PI-RADS category 4 or 5 lesions, in-bore biopsy demonstrated a significantly higher target-specific cancer detection rate than fusion biopsy. The odds of detecting any cancer were 2.28 times greater with in-bore biopsy. Although the difference in the likelihood of detecting ISUP grade group 2 or higher cancer did not reach statistical significance, it showed a trend favoring in-bore biopsy. However, when off-target sampling was considered, there was no significant difference in the detection rates between in-bore biopsy and combined fusion and systematic biopsy [8].

Another consideration revolves around the following question: whom should we subject to biopsy? Risk assessment involves evaluating various factors such as life expectancy, family history, risk calculators, digital rectal examination, PSA values, biomarkers, and results from multiparametric MRI.

Prostate cancer biomarkers play a crucial role in the diagnostic landscape, offering valuable insights into the assessment and management of the disease. Various biomarkers such as 4Kscore, Prostate Health Index (PHI), SelectMDx, ExoDx Intelliscore (EPI), and MyProstateScore (MPS) are available for comprehensive evaluation in prostate cancer diagnosis [1,2,4]. These biomarkers play a crucial role in improving risk stratification and management decisions by aiding in the detection and differentiation of clinically significant prostate cancer from insignificant cases [4,21].

Navigating the challenges associated with a negative multiparametric MRI poses a significant concern, as it raises questions about effectively managing cases where a biopsy is omitted despite the absence of abnormalities on the MRI. Emphasizing the importance of considering the negative predictive value in such scenarios—a metric indicating the likelihood that a negative MRI result corresponds to the absence of clinically significant prostate cancer.

In addressing this issue, two meta-analyses have provided valuable insights. Sathianathen et al. reported a negative predictive value of 90.8% for grade group (GG) 2 or higher disease and 97.1% for GG3 or higher disease [22]. Moldovan et al. similarly found a negative predictive value of 88.1% for GG2 or higher disease. These findings underscore the complexities in decision-making when dealing with negative MRI results and the need for a nuanced approach to ensure the accurate identification of clinically significant prostate cancer [23].

Transitioning to the discussion of two pivotal trials included in this review, the first highlighted is the PROMIS trial. PROMIS, a multi-center, paired-cohort study, aimed to evaluate the diagnostic accuracy of mpMRI and transrectal ultrasound-guided biopsy compared to a gold-standard reference template mapping biopsy. Inclusion criteria involved men (*n* = 576) with a PSA < 15 ng/mL and no previous biopsy history. Mapping biopsy results revealed that 71% of men had cancer, with 40% exhibiting clinically significant prostate cancer (Gleason score ≥ 4 + 3 or maximum cancer length ≥ 6 mm) [11].

In the context of clinically significant disease, mpMRI demonstrated higher sensitivity (93%) compared to transrectal ultrasound-guided biopsy (48%), though with lower specificity (41% for mpMRI; 96% for transrectal ultrasound-guided biopsy). These findings shed light on the comparative strengths and weaknesses of mpMRI and transrectal ultrasound-guided biopsy in diagnosing clinically significant prostate cancer.

Based on this information, implementing a triage mpMRI could potentially spare 25% of men from undergoing unnecessary prostate biopsies, simultaneously reducing the detection of clinically insignificant prostate cancer.

Turning attention to the second significant trial, the PRECISION trial enrolled 500 men with a clinical suspicion of prostate cancer who had not previously undergone a prostate biopsy. The trial compared MRI with or without a targeted biopsy against the standard transrectal ultrasound-guided biopsy. In the MRI group, men underwent a targeted biopsy if there was suspicion of prostate cancer on imaging and refrained from biopsy if the MRI yielded negative results. The primary outcome of this randomized clinical trial was the diagnosis of clinically significant prostate cancer. In the MRI-targeted biopsy group, 28% had a negative MRI and thus avoided biopsy. Among those undergoing targeted biopsy, 38% were diagnosed with clinically significant cancer, compared to 26% in the transrectal ultrasound-guided biopsy group (*p* = 0.005). Additionally, the MRI-targeted biopsy group exhibited fewer cases of clinically insignificant prostate cancer compared to the transrectal ultrasound-guided biopsy group [8].

Based on the aforementioned data, a negative MRI coupled with the decision to forgo a prostate biopsy results in an approximately 0–12% risk of overlooking clinically significant prostate cancer. Notably, the National Comprehensive Cancer Network (NCCN) guidelines strongly recommend mpMRI in cases of clinical suspicion of prostate cancer.

Exploring the significance of multiparametric MRI, it emerges as a pivotal screening tool with utility at various stages in the assessment of prostate cancer. Beyond offering exceptional anatomic and functional insights, the PI-RADS v2.1 standardizes acquisition, interpretation, and reporting procedures. Notably, multiparametric MRI findings enable targeted biopsy through in-bore, cognitive fusion, or MRI-US fusion techniques. In a landmark 2015 trial led by Siddiqui et al., MR/ultrasound fusion-guided biopsy was compared with ultrasound-guided biopsy in a cohort of 1003 men. The MR/ultrasound fusion biopsy diagnosed 30% more high-risk cancers and 17% fewer low-risk cancers compared to the standard biopsy. Combining standard biopsy cores with the targeted approach identified an additional 22% of mostly low-risk prostate cancer cases [24].

In 2021, Klotz et al. evaluated the noninferiority of MRI with targeted biopsy against systematic transrectal ultrasound-guided biopsies in detecting ISUP 2 or greater prostate cancer. The MRI-targeted biopsy approach was found to be non-inferior, with a better detection rate of ISUP > 2 disease and a lower detection of ISUP1 prostate cancer compared to the standard method [25].

Conclusively, MRI targeting enhances the detection of ISUP > 2 diseases in both initial and repeat biopsy settings while minimizing the detection of ISUP1 prostate cancer.

The landscape of prostate cancer screening has witnessed significant developments, with the 2022 NEJM GÖTEBORG-2 trial taking center stage in the quest for more effective methodologies. In this trial, spanning 37,887 men aged 50–60, participants were invited for a screening involving PSA and MRI, followed by targeted biopsy only. Of the 17,980 participants, 66 individuals (0.6%) in the experimental group were diagnosed with clinically insignificant prostate cancer, in contrast to 72 participants (1.2%) in the reference group. This marked a notable difference of −0.7 percentage points (95% CI −1.0 to −0.4), resulting in a relative risk of 0.46 (95% CI, 0.33 to 0.64). Additionally, the relative risk of clinically significant prostate cancer in the experimental group, when compared with the reference group, stood at 0.81 (95% CI, 0.60 to 1.1) [13].

The assessment of MRI for prostate biopsy, as summarized, unveils both advantages and considerations. On the positive side, directed biopsies, whether transrectal ultrasound-guided or transperineal, offer a targeted approach, enhancing the detection of clinically significant prostate cancer. There is potential for limiting the number of biopsies and even avoiding a prostate biopsy altogether.

However, certain challenges and drawbacks need acknowledgment. The process necessitates two separate visits, which may pose logistical challenges for some individuals. Patient factors, including size and anxiety, can impact the feasibility of this approach. Furthermore, the expense associated with MRI-based screening and the dependence on radiology expertise introduces practical considerations.

This strategy proves essential in reducing the risk of overdiagnosis and overtreatment of less aggressive prostate cancers, thus optimizing patient care and minimizing unnecessary biopsy procedures [26].

This clinical trial from Ahdoot investigated the efficacy of biopsies with MRI targeting in men with visible prostate lesions. Among 2103 participants undergoing both MRI-targeted and systematic biopsies, cancer was detected in 62.4%, and 19.2% proceeded to radical prostatectomy. Cancer detection rates varied between MRI-targeted and systematic biopsies for different grade groups, with combined biopsies leading to more diagnoses and grade upgrades. Solely relying on MRI-targeted biopsies would have misclassified 8.8% of clinically significant cancers (grade group ≥ 3). Among the subset of men undergoing radical prostatectomy, combined biopsy exhibited the fewest upgrades to grade group 3 or higher on histopathological analysis, with rates substantially lower compared to MRI-targeted biopsy and systematic biopsy. In conclusion, while combined biopsy enhanced overall cancer detection, MRI-targeted biopsy alone demonstrated limitations in accurately assessing the histologic grade of some tumors, emphasizing the nuanced considerations in prostate cancer diagnosis [2].

In essence, targeted biopsies offer a precision-driven alternative to the blanket approach of systematic biopsies, with the potential to significantly enhance diagnostic accuracy while sparing patients from unnecessary procedures.

### 4.1. Economic Considerations and Accessibility

The economic feasibility and accessibility of mpMRI and targeted biopsies vary across healthcare systems globally. While some countries with robust healthcare infrastructure may integrate these advanced diagnostic tools seamlessly, others may face challenges in terms of cost and accessibility, potentially limiting widespread adoption.

The economic accessibility of multi-parametric magnetic resonance imaging (mpMRI) for prostate cancer diagnosis is a topic of significant interest in healthcare. Several studies have explored the cost-effectiveness and implications of incorporating mpMRI into diagnostic pathways for prostate cancer.

While there are clear benefits to using mpMRI in prostate cancer diagnosis, concerns have been raised regarding the economic implications. The cost of MRI scanners, availability of trained personnel, and time taken per test are factors that could limit the widespread adoption of mpMRI for prostate cancer diagnosis. Conversely, in low- and middle-income countries, the economic feasibility of implementing mpMRI for prostate cancer diagnosis is more challenging. Limited resources, infrastructure, and expertise pose significant barriers to the widespread adoption of mpMRI in these regions.

### 4.2. Potential of Artificial Intelligence

Artificial intelligence (AI) is revolutionizing the field of prostate cancer diagnosis, particularly in the context of multiparametric MRI (mpMRI)-guided prostate biopsies. AI, including deep learning (DL), is being integrated into clinical practice to assist in therapeutic decisions and predict patient outcomes [27,28]. By using AI algorithms trained with the integration of MR-US image data and fusion biopsy trajectory-proven pathology data, it is possible to predict the volume and location of clinically significant cancer, aiding in the planning of focal therapy [28]. The utilization of AI in prostate MRI scans focuses on identifying cancer suspicious areas without requiring prior lesion information [29].

Studies have shown that AI can significantly improve the accuracy of detecting and differentiating clinically significant prostate cancer from insignificant cases, leading to better risk stratification and management decisions [29,30]. The combination of fusion magnetic resonance imaging-targeted biopsy with AI technology has been a significant breakthrough in enhancing the precision of prostate cancer diagnosis [31]. Moreover, MRI-ultrasound fusion biopsies have demonstrated improved detection of clinically significant prostate cancer compared to ultrasound-guided systematic biopsies alone [30].

Overall, AI in prostate cancer detection on imaging offers promising applications for various steps of the diagnostic pathway, including segmentation, lesion detection, and classification. While there are many encouraging results from research studies, further prospective multicenter trials are needed to fully understand the impact and utility of AI in improving radiologist performance and patient outcomes in prostate cancer diagnosis and management.

### 4.3. Advancements in Active Surveillance and Prostate Biopsy Strategies

In the realm of active surveillance for prostate cancer, the integration of multiparametric MRI and targeted biopsies has emerged as a pivotal aspect of refining patient management. It is essential to underscore that patients undergoing active surveillance should not entirely rely on an unchanged MRI status to forgo a biopsy. This caution stems from the recognized limitations of MRI, including its low sensitivity to predict progression, poor correlation with pathologic progression, and inadequate specificity to rule out high-grade disease in negative MRI results.

However, an intriguing development surfaces when MRI with targeted biopsy is employed before initiating active surveillance. This approach reduces the likelihood of discontinuing active surveillance within the first two years, with MRI-US fusion showcasing a notable advantage over systematic biopsy in detecting grade progression at the two-year mark.

While targeted biopsies have shown promise in diagnosing primary prostate cancer, their utility in identifying intraprostatic cancer recurrence after radiotherapy remains an area that warrants further investigation. The potential role of targeted biopsies in salvage focal ablation planning requires careful consideration and exploration, as highlighted by ongoing trials such as the FORECAST TRIAL [32].

## 5. Conclusions and Recommendations

The evolution of prostate cancer diagnosis towards targeted biopsy approaches, particularly those guided by mpMRI, marks a significant advancement in enhancing diagnostic accuracy and patient outcomes. It is imperative to continue refining these techniques, considering the patient-specific context and integrating the latest evidence into clinical practice, to further mitigate the risks associated with overdiagnosis and overtreatment.

### Recommendations

1. Patient-Centric Approach: Tailor biopsy approaches based on individual patient characteristics, risk factors, and preferences to optimize diagnostic precision and patient outcomes.

2. Incorporate Imaging Tools: Emphasize the continued importance of mpMRI in prostate cancer detection. Consider the use of MRI before a prostate biopsy, and contemplate targeted biopsy omission if the MRI yields negative results.

3. Optimize Biopsy Techniques:Acknowledge the strengths and limitations of both transrectal ultrasound-guided and transperineal biopsies. Prioritize the utilization of MRI-targeted biopsies to enhance diagnostic accuracy.

4. Combined Biopsies: Embrace the recommendation of performing combined systematic and targeted biopsies. This integrated approach harnesses the strengths of each method, offering a more comprehensive and accurate diagnosis.

5. Risk Assessment and Biomarkers:Evaluate risk factors, life expectancy, and family history, and utilize prostate cancer biomarkers in conjunction with imaging for a comprehensive risk assessment.

6. Follow Evidence-Based Guidelines: Adhere to established guidelines such as those provided by the European Association of Urology (EAU) for prostate biopsy, ensuring a standardized and evidence-based approach to diagnosis.

7. Active Surveillance Integration: Recognize the evolving role of mpMRI and targeted biopsies in active surveillance protocols. Implement targeted biopsies before initiating active surveillance to reduce the likelihood of discontinuation within the first two years.

## Figures and Tables

**Table 1:** Summary of studies’ characteristics.

Year	Reference	Number of Patients	Prior Negative Biopsy	Transrectal or Transperineal Biopsy	Median Age	Median PSA (ng/mL)	Median Prostate Volume (cc)	Cores per Target (MRI)	Comparator (Cores)	Definition of Clinically Significant Prostate Cancer	Detection Rate	Complications
2016	(Baco et al., 2016) [5]	175	Biopsy naive	TR	65	7.3	42	2	If a palpable and/or TRUS suspicious lesion was found, two targeted biopsies from the lesion were performed, followed by 12-core	MCCL > 5 mm for Gleason 6 or any MCCL for Gleason 7 disease	38%	
2016	(Pepe et al., 2017) [6]	200	saturation TP prostate biopsy	TR and TP	61	8.6		Targeted mpMRI/TRUS TR fusion guided biopsies (4 cores) and TP cognitive biopsies (4 cores)	//	Gleason score 6 and/or >2 positive cores	30%	None.
2017	(Porpiglia et al., 2017) [7]	107	Biopsy naive	TR	64	5.9	46.2	3–6	12	biopsy GS > 7 or MCCL > 5 mm	47%	
2018	(Kasivisvanathan et al., 2018) [8]	252	Biopsy naive	TR or TP route, according to local expertise	64.4	6.75	NR	4	12	>Gleason7 (3 + 4)	38%	Blood in the urine (30% vs. 63%), blood in the semen (32% vs. 60%), pain at the site of the procedure (13% vs. 23%), rectal bleeding (14% vs. 22%), and erectile dysfunction (11% vs. 16%). Two percent had serious adverse events.

## Data Availability

Data are available by request.
